# Enzymatic detergent reuse in gastroscope processing: a potential source of microorganism transmission*

**DOI:** 10.1590/1518-8345.3101.3211

**Published:** 2019-12-05

**Authors:** Maria Letícia de Miranda Mati, Natália Rocha Guimarães, Paula Prazeres Magalhães, Luiz de Macêdo Farias, Adriana Cristina de Oliveira

**Affiliations:** 1 Prefeitura Municipal de Belo Horizonte, URS Campos Sales, Belo Horizonte, MG, Brazil.; 2 Universidade Federal de Minas Gerais, Instituto de Ciências Biológicas, Belo Horizonte, MG, Brazil.; 3 Scholarship holder at the Coordenação de Aperfeiçoamento de Pessoal de Nível Superior (CAPES), Brazil.; 4 Universidade Federal de Minas Gerais, Escola de Enfermagem, Belo Horizonte, MG, Brazil.

**Keywords:** Patient Safety, Endoscopes, Detergents, Sanitizing Products, Microbiology, Infection Control, Segurança do Paciente, Endoscópios, Detergentes, Saneantes, Microbiologia, Controle de Infecção, Seguridad del Paciente, Endoscopios, Detergentes, Saneantes, Microbiología, Control de Infecciones

## Abstract

**Objective:**

to evaluate the potential contamination of enzymatic detergent from its reuse and to identify the microbiological profile in the solution used to clean gastrointestinal endoscopic devices.

**Method:**

cross-sectional study based on microbiological analysis of 76 aliquots of 19 different enzymatic detergent solutions used to clean endoscopic devices. The aliquots were homogenized, subjected to Millipore® 0.45 µm membrane filtration and the presumptive identification of microorganisms was performed by biochemical-physiological methods according to previously established specific bacterial groups that are of clinical and epidemiological relevance.

**Results:**

the mean values, as well as the standard deviation and the median, of the enzymatic detergent microbial load increased as the solution was reused. There was a significant difference between the means of after first use and after fifth reuse. A total of 97 microorganisms were identified, with predominance of the coagulase-negative *Staphylococcus, Pseudomonas* spp., *Klebsiella* spp., *Enterobacter* spp. genus, and *Escherichia coli* species.

**Conclusion:**

the reuse of the enzymatic detergent solution is a risk to the safe processing of endoscopic devices, evidenced by its contamination with pathogenic potential microorganisms, since the enzymatic detergent has no bactericidal property and can contribute as an important source for outbreaks in patients under such procedures.

## Abstract (pt)

**Objetivo:**

avaliar a potencial contaminação do detergente enzimático a partir de seu reuso e identificar o perfil microbiológico na solução utilizada na limpeza de aparelhos endoscópicos gastrointestinais.

**Método:**

estudo transversal realizado a partir da análise microbiológica de 76 alíquotas de 19 diferentes soluções de detergente enzimático utilizadas na limpeza de aparelhos endoscópicos. As alíquotas foram homogeneizadas, submetidas a filtração em membrana Millipore® 0,45µm e a identificação presuntiva dos microrganismos foi realizada por métodos bioquímico-fisiológico conforme grupos bacterianos específicos previamente estabelecidos e que apresentam importância clínica e epidemiológica.

**Resultados:**

Os valores médios, assim como o desvio-padrão e a mediana, da carga microbiana do detergente enzimático foram crescentes à medida que a solução foi reutilizada. Houve diferença significativa entre as médias de após primeiro uso e após quinto reuso. Foram identificados 97 microrganismos, com predomínio dos gêneros Staphylococcus coagulase negativa, Pseudomonas spp., Klebsiella spp., Enterobacter spp., e da espécie Escherichia coli.

**Conclusão:**

O reuso da solução de detergente enzimático constitui um risco ao processamento seguro dos aparelhos endoscópios, evidenciado pela sua contaminação com microrganismos que apresentam potencial patogênico, uma vez que o detergente enzimático não possui propriedade bactericida e pode contribuir como fonte importante para surtos em pacientes submetidos a tais procedimentos.

## Abstract (es)

**Objetivo:**

evaluar la potencial contaminación del detergente enzimático a partir de su reutilización e identificar el perfil microbiológico en la solución utilizada en la limpieza de aparatos endoscópicos gastrointestinales.

**Método:**

estudio transversal realizado a partir del análisis microbiológico de 76 alícuotas de 19 diferentes soluciones de detergente enzimático utilizadas en la limpieza de aparatos endoscópicos. Las alícuotas fueron homogeneizadas y sometidas a filtración en membrana Millipore® 0,45µm, la identificación presumida de los microorganismos fue realizada por métodos bioquímico-fisiológicos conforme grupos bacterianos específicos previamente establecidos y que presentan importancia clínica y epidemiológica.

**Resultados:**

los valores medios, así como la desviación estándar y la mediana, de la carga microbiana del detergente enzimático fueron crecientes a medida que la solución fue reutilizada. Hubo diferencia significativa entre las medias después del primer uso y después de la quinta reutilización. Fueron identificados 97 microorganismos, con predominio de los géneros *Staphylococcus* coagulase negativa, *Pseudomonas spp, Klebsiella spp, Enterobacter spp*, y de la especie *Escherichia coli.*

**Conclusión:**

la reutilización de la solución de detergente enzimático constituye un riesgo al procesamiento seguro de los aparatos endoscópicos, evidenciado por su contaminación con microorganismos que presentan potencial patogénico, ya que el detergente enzimático no posee propiedad bactericida y puede contribuir como fuente importante para brotes en pacientes sometidos a esos procedimientos.

## Introduction

Gastrointestinal endoscopic devices are equipment that cause great concern about processing. Used during the digestive endoscopy examination, these devices have external and internal surfaces exposed to high microbial load from their contact with the gastrointestinal tract, which makes proper decontamination necessary after each procedure to avoid cross contamination, to protect the team that reprocesses it against transmission of microorganisms and to prevent misdiagnosis, since biopsy fragments may remain inside the equipment and may be confused with those of another patient^(^ 1 ^-^ 3 ^)^.

Evidence suggests that the transmission of pathogens through endoscopic devices is an extremely rare event when the guidelines for processing these devices are respected^(^ 3 ^-^ 5 ^)^. The frequency of infection in digestive endoscopy is estimated to be 1 in 1.8 million procedures performed^(^ 1 ^)^. However, it is possible that this data underestimates the actual incidence of contamination since there are few estimates of infections resulting from these tests^(^ 3 ^,^ 6 ^)^.

However, when these infections occur, they can cause serious harm to the patient’s health, as they include serious diseases such as sepsis, bacteremia, pneumonia, gastroenteritis, and hepatitis B and C^(^ 6 ^-^ 7 ^)^. In 2013, in the United States, inadequate processing of endoscopic devices was responsible for the largest outbreak of carbapenem-resistant *Enterobacteriaceae* ever recorded. Patients who underwent endoscopic retrograde cholangiopancreatography (ERCP) were affected and during the period more than 18 deaths were reported^(^ 8 ^-^ 11 ^)^.

In order to achieve the utmost processing efficiency of endoscopic devices, it is important that both the process steps and the specific characteristics of the products used in cleaning these devices are complied.

In this context, nursing plays a central role as the processing of these devices is performed by them in most health services. It is the nursing professionals who, besides helping the endoscopic procedure, are responsible for the cleaning, disinfection, rinsing, storage of devices and preparation of sanitizing solutions^(^ 12 ^)^.

One of the products widely used in the processing of these equipment is the enzymatic detergent. Its function the breakdown of the organic matter of the devices after clinical use. Its reuse during cleaning of endoscopic devices is expressly warned by national and international institutions, associations and societies under penalty of efficiency loss^(^ 13 ^-^ 16 ^)^. However, it is known that in clinical practice this solution has been reused several times for immersion of different equipment.

This practice aims to reduce costs in endoscope processing without considering the impact of this reuse on the processing quality of devices, since enzymatic detergent has no bactericidal property^(^ 3 ^,^ 17 ^)^. There are few studies addressing the efficiency of the solution during its use in clinical practice, especially directly, as pointed sometimes that the reuse of enzymatic detergent may contribute to the recontamination of devices, compromising the action proposed by the solution^(^ 18 ^-^ 19 ^)^.

Given this finding, there is an important knowledge gap related to such a relevant aspect in the cleaning stage. Therefore, it is questioned: *Can the present microbial load and the microbiological profile recovered in the enzymatic detergent solution from its reuse during manual cleaning of gastrointestinal endoscopic devices compromise the effectiveness of processing?*

Thereby, this study was conducted in order to support evidence on the risk of reuse of enzymatic detergent solution, the consequential safety on endoscopic device processing, contributing to the improvement of nursing professionals’ knowledge regarding practices, adopted in cleaning endoscopic devices and assisting in the adequacy of institutional protocols and routines.

Thus, the objective was to evaluate the potential contamination of enzymatic detergent from its reuse and identify the microbiological profile in the solution used to clean gastrointestinal endoscopic devices.

## Method

Cross-sectional study conducted at the digestive endoscopy service of a public and general university hospital in Belo Horizonte, Minas Gerais, where the sample collection took place, and at the Oral and Anaerobic Microbiology Laboratory of the Institute of Biological Sciences of the Federal University of Minas Gerais, where the analysis of the collected materials took place. This study was approved by the Ethics and Research Committee of the National Health Council (Process: CAAE-67493417.1.0000.5149).

A total of 19 enzymatic detergent solutions used for cleaning endoscopic devices between October 2017 and June 2018 were analyzed. From each solution, four aliquots of enzymatic detergent were selected and collected before the first use in order to know the basal microbial profile of the solution, and after the first, third and fifth reuse, as a way to evaluate the possible changes in the microbiological profile along the different reuses. This way, 76 aliquots were analyzed.

The analysis interval of the samples was defined from a pilot study carried out for this purpose considering the difference between the recovery of microorganisms from the number of times the equipment would be immersed in the same solution of enzymatic detergent. Thus, we reached the analysis interval between one, three and five reuses of the solution in clinical practice.

The aliquots, whose volume was 10 mL, were collected directly from the container where the enzymatic detergent was stored (total volume of 20 liters). This material was stored in a sterile vial, refrigerated and transported to the laboratory.

For microbiological analysis the samples were homogenized in vortex for one minute and then 1 mL aliquots were inoculated into 9 mL Modified Letheem broth plus Tween 80. Subsequently, the samples were separately filtered on a sterile Millipore® membrane, with porosity of 0.45 µm and 47 mm in diameter.

The membranes were overlapped in Triptic Agar Soy (TSA) growth medium and incubated at 37°C for 24h to 48h. Representative colonies of different morphotypes were isolated in TSA and stored in a freezer at -80ºC in Brucella broth plus glycerol (10% final concentration) for future identification.

Gram staining was used for presumptive identification of microorganisms. Gram positive cocci were placed on Mannitol agar incubated at 37°C for 24 hours. Samples that showed growth on mannitol agar were subjected to biochemical tests of catalase, coagulase and DNAse. Gram negative rods were seeded in 1% glucose broth plus Andrade indicator and incubated at 37°C for 24 hours to separate glucose fermenting from non-fermenting microorganisms. Non-fermenting glucose microorganisms were placed on Cetrimide Agar at 37°C for 24 hours. Those who fermented glucose underwent modified Rugai tests.

For data analysis, descriptive statistics with frequency distribution and measures of central tendency were used. Statistical analyzes were performed considering the significance level of 5% (p-value = 0.05). The differences between the averages of the different reuses of the enzymatic detergent solution were evaluated by the Bonferroni test. In addition, the Mann Whitney test and nonparametric median tests were performed.

## Results

In the digestive endoscopy clinic, reusing the enzymatic detergent solution is usual, according to an internal protocol for its preparation and use per work shift, with an average duration of six hours, regardless of the number of times the equipment would be immersed in this period. Thus, the microbial load of 19 enzymatic detergent solutions was analyzed, respecting the previously established analysis interval and corresponding to the collection of an aliquot before use and an aliquot after the first, third and fifth reuse of the solution.

The descriptive analysis of the microbial load recovered in the enzymatic detergent solution during the different reuses is presented according to Table 1.

**Table 1:** Microbial charge recovered from enzymatic detergent solution after first, third and fifth use. Belo Horizonte, MG, Brazil, 2017-2018

Period	Microbial charge recovered in 1mL CFU/mL*			Microbial charge recovered in the total volume UFC^†^	
	Mean	Standard deviation	Median	Mean	Median
After first use	19.9	38.0	6	3.99x10^5^	1.20x10^5^
After third reuse	51.1	81.1	16	1.02x10^6^	3.20x10^5^
After fifth reuse	67.1	96.8	28	1.34x10^6^	5.60x10^5^

* CFU/mL = Colony Forming Unit per milliliter;

† CFU = Colony Forming Unit

It is observed that the mean values, as well as the standard deviation and the median, of the enzymatic detergent microbial load increased as the solution was reused.

Considering that the aliquots (1 mL) analyzed were part of an enzymatic detergent solution with a total volume of 20 liters, an average microbial load of 3.99x10^5^ CFU/solution was found in each container after the first use of the solution, averaging 1.34x10^6^ CFU in each solution after the fifth reuse. Considering the median, these values ​​correspond to 1.20x10^5^ and 5.60x10^5^ CFU, respectively.

Since this is a count, the microbial load distribution was not normal, which is why this variable was transformed using the natural logarithm function. Thus, the Bonferroni test only showed a significant difference between the means after the first use and after the fifth reuse (p-value 0.011), the other comparisons showed no significant difference. This result was confirmed by the nonparametric median tests (p-value 0.023) and Mann Whitney tests (p-value 0.004).

A total of 97 microorganisms were isolated. Gram negative corresponded to 78.4% (76/97), Gram negative rods were present in 44.7% of the aliquots (34/76), being *Pseudomonas* spp. the most frequently isolated microorganism (36.8%). Gram positive cocci were recovered in 15.8% of the aliquots (14/76), with *Staphylococcus* spp. negative coagulase present in 5.3% (4/76), according to Table 2.

**Table 2:** Microorganisms recovered from all enzyme detergent aliquots collected before use, after first use, after third and fifth reuse. Belo Horizonte, MG, Brazil, 2017-2018

Microorganisms recovered	%^†^	Frequência*				
		Before first use (n=19)	After first use (n=19)	After third use (n=19)	After fifth use (n=19)	All aliquots (n=76)
**Gram positive**	**16.5**	**5.3**	**36.8**	**15.8**	**10.5**	**17.1**
**Gram positive cocci**		**0**	**31.5**	**15.8**	**10.5**	**15.8**
*Staphylococcus* spp. negative coagulase		5.3	0	5.3	10.5	5.3
Unidentified		0	31.5	10.5	5.3	13.2
**Gram positive rods**		**0**	**5.3**	**0**	**0**	**2.6**
**Gram Negative**	**78.4**	**0**	**52.3**	**73.6**	**78.9**	**51.3**
**Gram Negative rods**		**0**	**47.4**	**73.6**	**78.9**	**44.7**
**Non-fermenting**						
*Pseudomonas* spp.		0	31.5	52.3	63.2	36.8
Unidentified		5.6	21.1	26.3	36.8	19.7
**Enterobacteria**						
*Enterobacter* spp.		0	0	5.3	0	1.3
*Escherichia coli*		0	0	5.3	5.3	2.6
*Klebsiella* spp.		0	0	5.3	0	1.3
Unidentified		0	10.5	10.5	10.5	7.9
**Gram negative cocci**		**0**	**5.3**	**0**	**5.3**	**2.6**
**Yeast**	**5.2**	**0**	**5.3**	**5.3**	**5.3**	**3.9**

* Frequency = Number of aliquots in which it was isolated/total number of aliquots of detergent;

† % = Percentage of all microorganisms isolated in enzymatic detergent aliquots

It was observed that as the cleaning solution was reused, Gram negative microorganisms, in special, *Pseudomonas* spp., were found more frequently, unlike Gram positive bacteria.

Only two samples collected before the use of enzymatic detergent showed microbial growth. In one of them, *Staphylococcus* spp. negative coagulase and, in another, an unidentified Gram-negative bacterium. Yeasts were recovered in three aliquots collected in different reuses of enzymatic detergent.

## Discussion

From the recovery of the average (or median) microbial load in the enzymatic detergent solution during product reuse, there was a progressive increase in contamination. Such result corresponds to what is expected from the enzymatic detergent solution, since it has no bactericidal action^(^ 10 ^,^ 17 ^)^. However, the number of microorganisms found in the enzymatic detergent container was extremely high, reaching an average of 13.41x10^6^ CFU in each solution after the fifth product reuse (5.60x10^5^ CFU, considering the median).

The reuse of the solution configures a source of transmission of microorganisms, since it can increase the microbial load of the endoscopic device and favor processing failures of this equipment. This aspect draws considerable attention when thinking that an endoscopic device may, after contact with the patient’s microbiota, present an average contamination of 10^9^ to 10^12^ CFU per equipment^(^ 20 ^)^.

In addition, the quantitative increase of microorganisms by the reuse of enzymatic detergent may make removal even more difficult during cleaning and thus favoring biofilm formation^(^ 21 ^)^. Studies show that cleaning and disinfection products commonly used in endoscopic device processing are not capable of removing biofilm in its entirety, which may allow the disintegration and transmission of microorganisms^(^ 22 ^-^ 24 ^)^. This is a matter of concern, since bacteria involved in biofilm formation may have up to 1,000 times greater resistance to antimicrobials compared to their suspension form^(^ 25 ^)^.

Regarding the types of contaminant microorganisms isolated in the enzymatic detergent solutions, Gram negative predominantly were detected, such as *Pseudomonas* spp., *Enterobacter* spp., *Escherichia coli* and *Klebsiella* spp. These findings are in line with the microorganisms present in the gastrointestinal tract microbiota, as well as the profile of microorganisms recovered in detergent formulations by other studies^(^ 26 ^-^ 27 ^)^. In addition, *Staphylococcus* spp. negative coagulase in 5.3% of the samples, as well as other unidentified Gram-positive cocci were found.

Gram positive rods have been isolated from aliquots from the cleaner solution and are likely to be due to environmental contamination since the enzymatic detergent storage container did not remain capped during use.

Although enterobacteria are part of the gastrointestinal tract microbiota, some species may be resistant to multiple antibiotics. Due to the severity of the infections caused and the limited therapeutic options, bacteria belonging to this family deserve attention in order to prevent outbreaks after endoscopic procedures^(^ 5 ^)^.

Among the identified enterobacteria, *Klebsiella* spp. was pointed out in 2013 as causing an outbreak that occurred in a German university hospital. At the time, this microorganism was isolated in 12 patients, six of them had undergone ERCP. The transmission of this microorganism was related to failures in the duodenoscopic processing used in the procedure^(^ 28 ^)^. The same happened between 2008 and 2009 in two hospitals in the United States, where *Klebsiella pneumoniae* was identified in seven patients who had also undergone ERCP. In these cases, all clones were resistant to Imipenem^(^ 29 ^)^.

*Pseudomonas* spp., a microorganism recovered in 63.3% of the aliquots referring to the fifth reuse of the enzymatic detergent solution, is an important cause of infections and outbreaks, especially in immunocompromised patients. It is the pathogen most commonly isolated in patients hospitalized for more than one week and is a frequent cause of healthcare-related infections^(^ 30 ^)^. *Pseudomonas aeruginosa* has been the most reported microorganism responsible for the transmission of infection during gastrointestinal endoscopy^(^ 5 ^)^.

Endoscope contamination by *P. aeruginosa* is mainly associated with equipment processing failures, to the quality of the water supplied to the endoscopy sector, and improper drying of the endoscope channels, since such microorganism has a preference for humid environments^(^ 1 ^,^ 31 ^-^ 32 ^)^. Infections following post endoscopy procedures due to contamination by *Pseudomonas* spp. include sepsis, cholangitis, abscesses, pancreatitis and pneumonia^(^ 5 ^)^.

In this study, before the first use of the enzymatic detergent solution, *Staphylococcus* spp. negative coagulase and non-fermenting Gram negative rods were recovered. Such findings are inferred from the potential contamination of the solution storage container, either through contamination of improper handling or cleaning. These findings corroborate studies that identified *Staphylococcus* spp. negative coagulase as the most frequently isolated microorganism (44.5%) in the hands of the evaluated health professionals^(^ 33 ^)^.

*Staphylococcus* spp. Negative coagulase was identified in 10.5% of the aliquots collected after the fifth reuse. These microorganisms are considered important pathogens in hospitalized and outpatients, and are among those most frequently isolated in clinical microbiology laboratories^(^ 34 ^)^. Species belonging to the genus have been associated with infectious processes in patients who have internal or implanted medical devices^(^ 34 ^-^ 35 ^)^.

Data collection from this study was performed in a single health service. However, it expresses the reality of most underdeveloped and developing countries, which do not have enough human and financial resources, impacting on the quality of practices related to the processing of health products, especially regarding to endoscopic devices. In this scenario, the reuse of enzymatic detergent is a high-risk reality for the processing of gastrointestinal endoscopes, as well as any other hospital medical article, as it contributes greatly to cleaning failure and is a potential source of outbreaks.

Considering that the enzymatic detergent solution aims to reduce the microbial load present in endoscopic devices from the breakdown of organic matter, the reuse promotes the reverse effect, causing contamination of these equipment, which configures a potential risk of failure. In case of outbreaks, when seeking to find the source of contamination of microorganisms, the right focus might not be given to reuse or misuse.

Outbreaks and their relationship to the use of health technologies, such as endoscopic procedures, have been drawing special attention after the American outbreak in 2015. Thus, in 2016, the Ecri Institute, a non-profit organization dedicated to researching the best resources for patient care, pointed out as first place in its annual ranking, the risk to patient safety when undergoing endoscopic procedures due to flaws from the cleaning and disinfection of these equipment. This concern remained a major focus of attention for the reports released in 2017 and 2018^(^ 36 ^)^.

Clearly, the processing of endoscopic equipment is a major challenge for services because of its complex design, numerous narrow channels, and for being a task with of a sequence spanning over 100 interconnected and dependent steps. All steps must be strictly followed, as failures in any of them can endanger the safety of the entire process^(^ 5 ^,^ 19 ^,^ 37 ^)^. The use of enzymatic detergent is only part of the cleaning step, however, when used incorrectly can negatively impact the whole process.

In this context, the role of nurses is extremely important. This professional is the one who should develop an institutional processing protocol and a verification tool related to the effectiveness of the established protocol. In addition, the nurse should be responsible for creating and managing an educational program presenting to the entire team to the importance of adhering to best practices throughout the process.

A limitation of this study can be attributed to the lack of information related to the susceptibility of the recovered microorganisms. However, preliminary analyzes did not show the presence of antibiotic resistant bacteria of last choice. However, the results express the reality experienced by health services, especially in underdeveloped and developing countries. Another limitation of the study is the difficulty of working with samples containing detergent, since the preservative present in the formulation inhibits bacterial growth and alters the viability of microorganisms. Research and tests are necessary to find the ideal neutralizer.

## Conclusion

The average microbial load recovered from enzymatic detergent solutions was increasing over reuse. Mean values ​​greater than 10^6^CFU per solution were verified from the third use. There was a significant difference between the means after first use and after fifth reuse (p-value 0.011).

Contamination by Gram positive microorganisms was always lower after the enzymatic detergent was reused, unlike Gram negative. *Pseudomonas* spp. the most frequently isolated microorganism.

Proper processing of endoscopic devices is a challenge for the nursing staff, given the difficulties inherent in the process due to the complex design of the equipment and the many steps to follow. This scenario can be considered even worse in underdeveloped and developing countries, where services are generally limited to essentially manual processing. In addition, the sectors generally have a nursing staff with fewer professionals than necessary, and work overload may contribute to insufficient adherence to established protocols.

There are numerous guidelines and steps that the endoscopic device must undergo in order to achieve effective processing. All recommendations must be periodically reviewed by the processing team, as each action taken can strongly impact the results to be achieved at the end of the process, especially when not done or poorly performed. Further research aimed at quantitative analysis of microbial load present in endoscopic apparatus before and after immersion in reused enzymatic detergent compared to unused detergent is suggested to consolidate the results presented in this study.
